# Polycystic ovary syndrome and thyroid disorder: a comprehensive narrative review of the literature

**DOI:** 10.3389/fendo.2023.1251866

**Published:** 2023-08-11

**Authors:** Stefano Palomba, Carla Colombo, Andrea Busnelli, Donatella Caserta, Giovanni Vitale

**Affiliations:** ^1^ Division of Gynecology, Sant’Andrea Hospital, University “Sapienza” of Rome, Rome, Italy; ^2^ Division of Endocrine and Metabolic Diseases, Istituto Auxologico Italiano IRCCS, Milan, Italy; ^3^ Department of Pathophysiology and Transplantation, University of Milan, Milan, Italy; ^4^ Department of Gynecology, Division of Gynecology and Reproductive Medicine, Fertility Center, Humanitas Clinical and Research Center-IRCCS, Rozzano, Milan, Italy; ^5^ Department of Biomedical Sciences, Humanitas University, Milan, Italy; ^6^ Department of Medical Biotechnology and Translational Medicine (BIOMETRA), University of Milan, Milan, Italy; ^7^ Laboratory of Geriatric and Oncologic Neuroendocrinology Research, Istituto Auxologico Italiano, IRCCS, Milan, Italy

**Keywords:** endocrine disease, infertility, polycystic ovary syndrome, PCOS, review, thyroid

## Abstract

**Background:**

Published data on the relationship between polycystic ovary syndrome (PCOS) and thyroid dysfunction are sparse and confusing.

**Objective:**

To comprehensively review data available in the literature regarding the relationship between PCOS and the thyroid function, and its abnormalities.

**Methods:**

Nine main areas of interest were identified and analyzed according to the available evidence: 1) Evaluation of thyroid function for PCOS diagnosis; 2) Epidemiology data on thyroid function/disorders in patients with PCOS, and vice versa; 3) Experimental data supporting the relationship between thyroid function/disorders and PCOS; 4) Effects of thyroid function/disorders on PCOS features, and vice versa; 5) Effect of thyroid alterations on the cardiometabolic risk in women with PCOS; 6) Effect of thyroid abnormalities on reproductive outcomes in women with PCOS; 7) Relationship between thyroid function/abnormalities in patients with PCOS who are undergoing fertility treatment; 8) Effect of treatments for thyroid diseases on PCOS; and 9) Effect of treatments for PCOS on thyroid function. An extensive literature search for specific keywords was performed for articles published from 1970 to March 2023 using PubMed and Web of Science. Data were reported in a narrative fashion.

**Results:**

PCOS is a diagnosis of exclusion for which diagnosis is possible only after excluding disorders that mimic the PCOS phenotype, including thyroid dysfunctions. However, the tests and the cutoff values used for this are not specified. Many experimental and clinical data suggest a relationship between perturbations of the thyroid function and PCOS. Direct and unequivocal evidence on the effects of thyroid function/disorders on PCOS features are lacking. High thyroid-stimulating hormone levels and subclinical hypothyroidism may be associated with significant worsening of several intermediate endpoints of cardiometabolic risk in women with PCOS. Thyroid abnormalities may worsen reproductive outcomes, especially in patients undergoing fertility treatment. To date, there are no data demonstrating the efficacy of thyroid medications on fertility and cardiometabolic risk in women with PCOS. Lifestyle modification changes, metformin, and vitamin D seem to improve thyroid function in the general population.

**Conclusion:**

PCOS and thyroid disorders are closely related, and their coexistence may identify patients with a higher reproductive and metabolic risk. Regular screening for thyroid function and thyroid-specific autoantibodies in women with PCOS, particularly before and during pregnancy, is highly recommended.

## 1 Introduction

Polycystic ovary syndrome (PCOS) is a complex and heterogeneous disease, and the most common endocrine disorder among women who are at reproductive age, with a prevalence that is widely variable according to geographic areas and the diagnostic criteria adopted (1). Even though the diagnostic criteria defined during the Rotterdam consensus workshop in 2003 (2) were initially criticized, expert opinions and clinical guidelines subsequently confirmed their value (1, 3, 4). These diagnostic criteria consist of the combination of at least two of three of the following features: oligo- and/or anovulation, clinical and/or biochemical signs of hyperandrogenism, and polycystic ovarian morphology (PCOM). More recently, both the American College of Obstetricians and Gynecologists (5) and the International PCOS Network (6) used the same original criteria, with only a few differences.

Thyroid disorders may also cause menstrual dysfunction, infertility, and metabolic disorders, and are extremely common in females (7–9). Although data on thyroid function/dysfunction in women with PCOS are sparse and confusing, growing evidence suggests a potential link between these diseases (10, 11). Genetic susceptibility to the onset of both diseases is a potential mechanism of association between thyroid disease and PCOS, but a common genetic profile has not yet been found. Furthermore, it was hypothesized that altered estrogen/androgen balance in women with PCOS may predispose these patients to hypothyroidism, but these data are inconclusive. Further extensive studies in these fields are needed to assess which mechanisms cause this correlation.

The aim of this article is to comprehensively review, in a narrative fashion, data available on thyroid function/dysfunction in women with PCOS, and to clarify the relationship between these two common medical conditions.

## 2 Methods

An extensive literature search was performed for articles published from 1970 to March 2023 using PubMed and Web of Science. Specific keywords used in the search were: “thyroid” and/or “hypothyroidism” and/or “hyperthyroidism” and/or “autoimmune thyroiditis” and/or “Hashimoto’s thyroiditis” and/or “chronic lymphocytic thyroiditis” and/or “Grave’s disease” and/or “TSH” with “polycystic ovary syndrome” and/or “polycystic ovary disease” and/or “PCOS.” Subsequently, the same search terms were paired with terms covering specific features/characteristics related to PCOS, such as “polycystic ovaries,” “oligo-amenorrhea,” “oligo-anovulation,” “ovulatory dysfunction,” “chronic anovulation,” “amenorrhea,” “oligo-amenorrhea,” “hyperandrogenism,” ‘testosterone,” “anti-Mullerian hormone,” “AMH,” “hyperestrogenism,” “estrogens,” “estradiol,” “hyperinsulinemia,” “insulin resistance,” “hirsutism,” “inflammation,” “body mass index,” “BMI,” “obesity,” “infertility,” “sterility,” “diabetes,” and “autoimmunity.”

The title and abstract of all articles on the relationship between PCOS and thyroid function were independently screened by all authors without language restriction. Full texts of eligible articles were then selected, and only articles considered relevant by the authors were cited and discussed. Additional journal articles were identified from the bibliographies of included studies. Specific inclusion and exclusion criteria to identify, select, and include in the final analysis of the articles were not predefined at the start of this narrative review.

## 3 Results

Following extensive revision of the literature, each author proposed a list of potential and specific topics to analyze. After discussion, all authors agreed on the main issues to evaluate (**Table 1**).

**Table 1:** Selected issues on the relationship between PCOS and thyroid function/disorder, reported as scientific questions to answer.

1. Is the evaluation of the thyroid function for a diagnosis of PCOS needed, and which clinical and biochemical parameters should be assessed?
2. Are there any epidemiological data linking thyroid disorders and PCOS?
3. Are there experimental data to support the relationship between thyroid function and PCOS?
4. What is the effect of thyroid function/dysfunction on PCOS features, and vice versa?
5. What is the effect of thyroid alterations on cardiometabolic risk in women with PCOS?
6. What is the effect of thyroid abnormalities on reproductive outcomes in women with PCOS?
7. Does thyroid function/dysfunction influence reproductive outcomes in patients with PCOS undergoing fertility treatment?
8. What is the effect of treatments for thyroid diseases on PCOS?
9. What is the effect of treatments for PCOS on thyroid function?

PCOS, polycystic ovary syndrome.

### 3.1 Evaluation of thyroid function for PCOS diagnosis

In all main articles in which different diagnostic criteria were proposed (2, 12, 13), it was emphasized that PCOS is a diagnosis of exclusion, where diagnosis is only possible after excluding disorders that mimic the PCOS phenotype. In the original published manuscript from the Rotterdam consensus workshop (2), the need to exclude hyperandrogenic diseases, such as congenital adrenal hyperplasias, androgen-secreting tumors, and Cushing’s syndrome was specified, but limited value was given to the exclusion of thyroid dysfunctions and hyperprolactinaemia due to their low incidence among patients with PCOS. However, because of the high frequency of thyroid disease in women with menstrual disorders, the assessment of thyroid function, together with the exclusion of other diseases, including hyperprolactinemia, acromegaly, genetic defects in insulin action, primary hypothalamic amenorrhea, primary ovarian failure, and syndrome of severe insulin resistance was recommended in other articles (1, 3–6, 13).

**Table 2** summarizes the different recommendations from consensus conferences and scientific societies to assess thyroid function in patients with suspected PCOS. It is noteworthy that this issue was discussed and adequately referenced in a few manuscripts only. The Endocrine Society practice guideline (3) specifies that serum thyroid stimulating hormone (TSH) levels higher than the upper limit of normal suggests that the patient has hypothyroidism and a TSH level lower than the inferior limit suggests that the patient has hyperthyroidism. However, the Scientific Statement of experts in PCOS (1) and clinical guidelines from the American Association of Clinical Endocrinologists (AACE), American College of Endocrinology (ACE), and Androgen Excess and PCOS Society (AES) (4) only include a few lines on the need to exclude endocrinopathies, including thyroid abnormalities, when PCOS is suspected. No specific recommendation regarding this issue was given in the more recent guideline from the International PCOS Network (6).

**Table 2:** Recommendations from consensus conferences and scientific societies on the assessment of thyroid function in patients with suspected PCOS.

Reference	Article type	Exclusion of thyroid disease	Clinical assessment	Biochemical assessment	Reason given to screen/not screen thyroid disease	Comments
Zawadski and Dunaif, (12)	Textbook from NIH meeting	No	Any	Any	————————–	———————–
Rotterdam ESHRE/ASRM-Sponsored PCOS consensus workshop group, (2)	ESHRE/ASRM consensus conference	No	Any	Any	Low incidence of thyroid disease in women with PCOS	Routine TSH assay in hyperandrogenic patients should be not discouraged
Azziz et al., (13)	AES consensus conference on PCOS diagnosis	Yes, but not mandatory	Any	Any	Thyroid abnormalities may cause ovulatory dysfunction despite a low prevalence	Hypo- and hyperthyroidism may influence PCOS diagnosis, and screening of patients with suspected PCOS for thyroid dysfunction may be cost-effective in asymptomatic patients with thyroid dysfunction
Legro et al., (3)	ES clinical guideline	Yes, but only suggested	Any	Serum TSH	Thyroid disease may present in women with irregular menstrual cycles	Suggestion for screening is based on low quality evidence (reinforced in case of amenorrhea and/or severe phenotypes). Serum TSH levels higher than the upper limit of normal suggests hypothyroidism; serum TSH levels lower than the inferior limit (<0.1 mIU/L) suggest hyperthyroidism
Goodman et al., (4)	AACE, ACE, and AES Society clinical guidelines	Yes	Any	Any	No motivation	Usefulness of the serum 17OHP and AMH assays discussed
Dumesic et al., (1)	Scientific statement of experts in PCOS	Yes	Any	Any	No motivation	Only a few lines in the text on excluding other endocrinopathies
Teede et al., (6)	International PCOS Network evidence-based guideline	Any	Any	Any	No motivation	Exclusion of other endocrinopathies for PCOS diagnosis not assessed/discussed
ACOG Committee on Practice Bulletins, (5)	ACOG clinical guidelines	Yes	Any	Serum TSH	High frequency in women with menstrual disorders	———————–

17OHP, 17-hydroxyprogesterone; AACE, American Association of Clinical Endocrinologists; ACE, American College of Endocrinology; ACOG, American College of Obstetricians and Gynecologists; AES, Androgen Excess and PCOS Society; AMH, anti-Mullerian hormone; ASRM, American Society for Reproductive Medicine; ES, Endocrine Society; ESHRE, European Society of Human Reproduction and Embryology; NIH, National Institute of Health; PCOS, polycystic ovary syndrome; TSH, thyroid stimulating hormone.

In conclusion, available data from published articles on the diagnostic criteria for PCOS diagnosis suggest excluding thyroid diseases but do not report on the specific thyroid diseases to exclude, tests needed for a PCOS diagnosis, or specific cutoff values for those tests to use.

### 3.2 Epidemiological studies on thyroid disorders in patients with PCOS, and vice versa

The presence of thyroid diseases is an exclusion criterion for a PCOS diagnosis. Thus, the prevalence of clinical thyroid dysfunctions, i.e., hyper- or hypothyroidism should be zero in the PCOS population. However, prevalence data on PCOS features in patients with hyper- or hypothyroidism may be interesting to explore. A literature search for epidemiological studies on thyroid disorders in patients with PCOS, including autoimmune thyroiditis (AIT), subclinical hypothyroidism (SCH), Graves’ disease (GD), thyrotoxicosis, thyroid nodules, nodular goiter, and thyroid malignancy was performed. Many of those studies discussed the AIT presence, but data regarding other thyroid disorders were sparse. **Table 3** summarizes the main data found (discussed further, below).

**Table 3:** Main clinical studies on potential correlations between thyroid disease and PCOS.

References	Country	Study design	Patients studied (n.)	Aim	Conclusions
AIT and PCOS					
Du and Li, (14)	China	Meta-analysis	1605 (726 PCOS patients vs 879 patients without PCOS)	To assess the relationship between PCOS and thyroiditis	Prevalence of AIT in patients with PCOS is significantly higher than in patients without PCOS. Data suggest that PCOS may be associated with AIT
Romitti et al., (15)	Brazil	Meta-analysis	2197 (1210 PCOS patients vs 987 patients without PCOS)	To estimate AIT prevalence and risk in women with PCOS	Meta-analysis provides evidence of higher AIT prevalence in patients with PCOS compared with healthy patients. Physicians should consider screening for thyroid function and thyroid-specific autoantibodies at PCOS diagnosis, even in the absence of symptoms related with thyroid dysfunction
Ho et al., (16)	China	Cohort	33655 (cases: 6731 women with AIT (5399 GD and 1332 HT); 26924 healthy women	To investigate PCOS prevalence and its comorbidities in patients with AIT	Findings support the established common mechanism between PCOS and AIT
Kim et al. (17)	Korea	Case-control	553 (210 PCOS patients vs 343 healthy women)	To assess the prevalence of anti-TPO Ab and hypoechoic USG in women with PCOS	AIT is not more prevalent in women with PCOS vs women without PCOS. However, among women with PCOS, patients with AIT have a significantly higher adiposity and insulin resistance index vs those without AIT
SCH and PCOS					
Ding et al., (18)	China	Meta-analysis	1232 (692 patients with PCOS vs 540 patients without PCOS)	To evaluate SCH prevalence in women with PCOS	SCH risk is higher in women with PCOS vs women without PCOS
Zhang et al., (19)	China	Cohort	34 obese patients with SHC vs obese patients without SCH	To determine whether SCH increases prevalence of PCOS	PCOS frequency does not differ between the two groups (56.1% for normal thyroid function vs 60.2% for SCH)
Kamrul-Hasan et al., (20)	Bangladesh	Case-control	465 (50 PCOS patients vs 415 patients without PCOS)	To assess if SCH in women with PCOS is a metabolic/reproductive risk factor	The similar adverse reproductive and metabolic consequences in women with PCOS with/without SCH indicates that these consequences are due to PCOS alone; the additional presence of SCH in these patients may not impart additional risks
Raj et al., (21)	Pakistan	Case-control	400 (200 patients with PCOS vs 200 patients without PCOS)	To assess if SCH is more frequent in women with PCOS vs healthy women	Data suggest a strong association of SCH in women with PCOS vs healthy women
Xu et al. (19)	China	Cohort	3189 (594 patients with PCOS vs 2595 patients without PCOS)	To evaluate the effect of TSH on IVF outcomes	TSH level in patients with PCOS with normal thyroid function is higher than that in patients without PCOS, and is negatively correlated with the oocyte maturation in IVF
Gawron et al., (22)	Poland	Cohort	367 women with PCOS	To evaluate whether SCH with/without anti-thyroid Ab impacts on the PCOS phenotype and alters biochemical/clinical parameters	SCH alters metabolic, but not hormonal, parameters in PCOS. Among all parameters studied, the strongest relationship with SCH is confirmed for insulin resistance and dyslipidaemia.
Rojhani et al., (23)	Iran		851 (207 PCOS patients vs 644 patients without PCOS)	To assess whether there is a difference between PCOS and control groups in terms of the upper reference limit of TSH and to identify SCH prevalence in women with PCOS vs women without PCOS	SCH prevalence and the upper reference limit of TSH are not significantly different in patients with PCOS vs patients without PCOS
GD and PCOS					
Glintborg et al., (24)	Denmark	Cohort	73223 (18476 patients with PCOS vs 54757 healthy women)	To investigate risk of thyroid disease in Danish women with PCOS	Findings highlight the importance of screening for thyroid disease at the time of PCOS diagnosis and during patient follow-up
Chen et al., (25)	Taiwan	Cohort	16197 (5399 women with GD vs 10798 women without GD)	To assess whether GD is a risk factor for developing PCOS	Women with GD are at a risk of developing PCOS. A higher incidence of comorbidities, including hyperlipidaemia, is noted in women with GD and PCOS
Botello et al., (26)	Colombia	Meta-analysis	47509 patients with thyroid autoimmunity	To determine the prevalence of these types of polyautoimmunity in patients with AIT as the index condition	Latent and overt polyautoimmunity are common in patients with AIT
Goiter/thyroid nodules and PCOS					
Duran et al., (27)	Turkey	Case-control	133 (70 PCOS patients vs 60 healthy women)	Estimate the frequency of nodular goiter in patients with PCOS	It is not possible to demonstrate a significant relation between thyroid volume, thyroid nodule, and AIT frequency in patients with/without PCOS. The increased frequency of goiter and AIT in patients with PCOS may be related to a component of metabolic syndrome rather than the PCOS diagnosis
Glintborg et al., (24)	Denmark	Cohort	18476 (1146 PCOS patients vs 54757 women without PCOS)	To investigate risk of thyroid disease in Danish women with PCOS	Data demonstrate a risk of developing a goiter that is significantly higher in women with PCOS vs healthy controls

AIT, autoimmune thyroid disease; anti-TPO Ab, anti-thyroid peroxidase antibody; CI, confidence interval; GD, Graves’ disease; HT, hypothyroidism; IVF, in vitro fertilization; OR, odds ratio; PCOS, polycystic ovary syndrome; SCH, subclinical hypothyroidism; TSH, thyroid stimulating hormone; USG, ultrasonography.

#### 3.2.1 AIT and PCOS

AIT, also known as Hashimoto’s thyroiditis (HT) or chronic lymphocytic thyroiditis, is the most common cause of hypothyroidism (28), characterized by thyroid infiltration by inflammatory cells and the presence of antithyroglobulin antibodies (anti-TG Ab) or anti-thyroid peroxidase antibodies (anti-TPO Ab). Thyroid autoantibodies reflect the general activation of the immune system and substantially contribute to the pathogenesis of AIT by activating complement/antibody-dependent cellular cytotoxicity leading to apoptosis of thyroid cells and the gradual destruction of follicles in the thyroid gland. AIT diagnosis is generally based on increased levels of anti-TPO Ab (with/without increased levels of anti-TG Ab) and is associated with the presence of diffuse/irregular hypoechogenicity of the thyroid gland during ultrasound evaluation.

A systematic review with meta-analysis (14) that aimed to study the relationship between PCOS and AIT was published in 2013. In this article, six studies that included 726 women with PCOS, diagnosed according to different criteria (including Rotterdam, National Institutes of Health [NIH]), or other criteria), and 879 patients without PCOS were analyzed. The prevalence of AIT was 22.8% and 5.7% in PCOS and non-PCOS groups, respectively. AIT was about fivefold higher in patients with PCOS versus patients without PCOS (odds ratio [OR] 4.81, 95% confidence interval [CI] 2.88−8.04). Mean TSH levels were slightly higher in patients with PCOS vs patients without PCOS; this increase in mean TSH level was statistically significant, with a mean difference of 0.62 (95% CI 0.21−1.02) (14). Anti-TPO Ab-positive patients were about threefold higher among patients with PCOS versus patients without PCOS (OR 3.32, 95% CI 1.25 to 8.87) and anti-TG Ab-positive patients were about twofold higher among patients with PCOS versus patients without PCOS (OR 1.93, 95% CI 1.23 - 3.02) (14). The goiter presence (OR 3.36, 95% CI 2.14 - 5.26) and a hypoechoic ultrasound pattern of the thyroid (OR 4.11, 95% CI 2.17 to 7.82) were more frequent in women with PCOS versus those without PCOS, and the thyroid volume of patients with PCOS was larger than that of control patients (14) (**Table 3**).

Another systematic review, including data until August 2017, confirmed the increased incidence of AIT in women with PCOS (15). The analysis included 13 studies, 1210 women with PCOS diagnosed according to Rotterdam criteria, and 987 healthy controls. AIT prevalence was 26% and 9.7% in patients with PCOS and the control group, respectively. The risk of developing AIT in women with PCOS was more than threefold higher versus the control group (OR 3.27, 95% CI 2.32−4.63). Interestingly, data from analysis of different geographical regions confirmed these findings in patients with PCOS in different geographic regions. The risk of developing AIT was four times higher in Asian (28.9%) patients with PCOS versus Asian patients without PCOS (8.6%; OR 4.56, 95% CI 2.47−8.43) but lower by less than twofold for South American patients with PCOS (26.6%) versus South American patients without PCOS (20.5%; OR 1.86, 95% CI 1.05−3.29). An intermediate risk (about threefold higher) was detected for European patients with PCOS (21.8%) versus European patients without PCOS (7.8%; OR 3.27, 95% CI 2.07 −5.15) (15). Findings for serum TSH levels were heterogeneous; about one-half of the studies reported that TSH levels were significantly higher in women with PCOS versus control groups, whereas other studies either found no difference in TSH levels for patients with/without PCOS or did not evaluate this (**Table 3**). Moreover, a recent case-control study (17) showed no difference in AIT prevalence in 210 women with PCOS versus 343 patients without PCOS. However, among women with PCOS, patients with AIT had a significantly higher adiposity and insulin resistance index versus those without AIT (17). A very large cohort study (16) evaluated PCOS prevalence and its comorbidities in patients with AIT. The analysis of 33655 patients with AIT and 26924 patients without AIT demonstrated an increased risk of PCOS in patients with AIT versus patients without AIT (adjusted hazard ratio [aHR] 1.39, 95% CI 1.07−1.71) (16).

Finally, a recent systematic review (29) including 20 studies and 7857 participants confirmed that women with PCOS have an increased risk of AIT versus patients without PCOS, and AIT prevalence was higher in South America versus Asia/Europe. This systematic review also showed that patients with AIT have an increased risk of developing PCOS versus patients without AIT, and PCOS prevalence was higher in India and Turkey versus other countries.

#### 3.2.2 SCH and PCOS

SCH is defined as the presence of elevated levels of TSH with normal free thyroxine levels (30). The cutoff value of TSH to determine the presence of SCH is not unequivocal throughout published studies (31, 32) (**Table 3**).

SCH prevalence in women with PCOS varies from 10−25% (33), whereas the prevalence of SCH and clinical hypothyroidism in women of reproductive age is 4−10 and 0.1−2%, respectively (34). Thus, SCH in women with PCOS may be at least twofold higher than SCH in unselected women (21). A recent study (23) on 207 women with PCOS and 644 healthy controls showed no difference in 95 percentiles of TSH concentrations and no association between PCOS status and SCH (aOR 1.40, 95% CI 0.79−2.50).

Discrepancies in SCH prevalence in women with PCOS may be due not only to the different cutoff TSH values used (18) but also to the different criteria for PCOS diagnosis or distinct PCOS phenotypes (including non-hyperandrogenic, ovulatory, non-PCOM, classical, PCOS meeting NIH criteria, or the full-blown/complete phenotypes). Albeit serum TSH levels were not associated with PCOS features, including hyperandrogenism, ovulatory dysfunction, and PCOM, a significant relationship between PCOS phenotype and TSH levels was detected (35). As TSH level increased, the proportion of women with complete PCOS phenotype (i.e., hyperandrogenism plus ovulatory dysfunction plus PCOM) increased, while the proportion of patients with the non-PCOM or non-hyperandrogenic phenotype decreased (35). No effect was observed on the proportion of women with women with the ovulatory phenotype (35). A meta-analysis of 6 studies (18) including 692 patients with PCOS and 540 patients without PCOS reported a threefold higher risk of SCH in women with PCOS versus patients without PCOS (OR 2.87, 95% CI 1.82−9.92). Further analysis of the data to only include studies in which a SCH diagnosis was ascertained using a TSH cutoff of ≥4 mUI/L, the SCH risk was 3.59 times higher in women with PCOS versus women without PCOS (18). Serum TSH concentration was higher in an infertile population of euthyroid women with PCOS versus euthyroid women without PCOS (36).

However, other authors reported no significant association of any PCOS phenotype with SCH with/without AIT, and no significant difference in hormonal parameters and the modified Ferriman-Gallwey scale score in women with PCOS with/without SCH (22). The reported rate of SCH with/without AIT is also extremely variable. In women with PCOS, the reported SCH prevalence was lower than AIT prevalence in one publication and higher than AIT prevalence in another publication (20, 22), suggesting a potentially different pathogenesis for SCH in patients with PCOS. Data on PCOS prevalence in patients with SCH are limited. A cohort study of 534 obese women demonstrated that the rate of PCOS diagnosis between patients with SCH (60.2%) and patients without SCH (56.1%) was not significant. This finding was also confirmed after adjusting the data for confounding factors (aOR 0.9, 95% CI 0.6−1.7) (19).

#### 3.2.3 GD and PCOS

GD is defined as an autoimmune disease resulting in the overproduction of thyroid hormones and hyperthyroidism/thyrotoxicosis (37). While autoimmune hypothyroidism is relatively frequent in women of childbearing age, GD is less frequent, and this influences the availability of correlation studies. In geographical regions without a high prevalence of iodine deficiency, the lifetime risk is 3% for women and 0.5% for men, with an estimated prevalence of 0.5−2% in females (38).

Data from a very large Danish cohort study (24) confirmed that the risk of developing thyroid disease for women with PCOS was more than double that for an age-matched control population (HR 2.5, 95% CI 2.3−2.7), with an incidence rate for thyrotoxicosis of 1.4 per 1000 patients/year for patients with PCOS versus 0.5 per 1000 patients/year for patients without PCOS. However, this risk was lower than the risk of hypothyroidism (24).

Although recent meta-analysis data showed that GD was the most common type of thyroid autoimmunity in patients with AIT (26), correlation studies on GD in women with PCOS, and vice versa, were limited. These data were reported in a few case reports and one cohort study that included a large number of patients (16197 patients of whom 5399 were women with GD and 10798 were patients without GD) (25). The cumulative incidence of PCOS was significantly higher in patients with GD versus patients without GD (aHR 1.47, 95% CI 1.09−1.98), suggesting that GD is a risk factor for developing PCOS (25).

In conclusion, data on the association between GD and PCOS are extremely limited. More extensive multicenter cohort studies are needed for further insight into the potential association between these diseases.

#### 3.2.4 Thyroid nodules and PCOS

Specific data on the presence of thyroid nodules in women with PCOS are sparse and confounded with other thyroid alterations. A retrospective study (39) on 178 patients with PCOS and 92 body mass index (BMI)-matched control patients showed that the number of thyroid nodules of ≥1 cm was higher in women with PCOS versus the control group. Further analysis of the PCOS phenotypes suggested that women with a complete PCOS phenotype were more often affected by thyroid nodules (39). Another study with a small sample size (40) showed a close association between thyroid nodules and ovarian volume in women with PCOS.

In conclusion, considering previous data and risk factors for developing thyroid nodules (41), including the female sex and metabolic syndrome, it is possible to hypothesize an increased prevalence of thyroid nodules in women with severe PCOS phenotypes and metabolic comorbidities. However, the exact prevalence of this cannot be calculated and further investigations involving larger cohorts of patients are required to establish the validity of this association.

#### 3.2.5 Goiter and PCOS

Regarding the prevalence of nodular goiter in women with PCOS, available data are also extremely limited, although the presence of thyroid nodules is relatively frequent in fertile young patients (42).

In a case-control study involving a small number of patients (27), no significant relationship was found between thyroid volume and nodular goiter prevalence in 70 women with PCOS versus 60 women without PCOS (27). However, this study also demonstrated no increase in the incidence of AIT in women with PCOS (27). More recently, a large cohort study demonstrated that the risk of developing a goiter was significantly higher in women with PCOS versus women without PCOS (OR 2.0, 95% CI 1.1−3.4) (24). The incidence of goiter in that study was 1.4 patients/year in women with PCOS versus 0.7 patients/year in the control population (24) confirming the complex nature of hormonal interactions in women with PCOS (43).

#### 3.2.6 Thyroid cancer and PCOS

Thyroid cancer is the most prevalent endocrine malignancy, and its incidence is more than threefold higher in women versus men (44). Approximately three quarters of thyroid cancers are diagnosed in the women (44).

The relationship between thyroid cancer and PCOS is still unknown and published data on this are limited. Available data frequently discuss the incidence of thyroid nodules instead of thyroid cancer in women with PCOS and indirect evidence for a potential link between thyroid cancer and PCOS. Direct evidence from epidemiological studies on the risk of thyroid cancer in patients with PCOS is not currently available in the literature.

### 3.3 Experimental data on the relationship between thyroid function and PCOS

Sparse and heterogeneous experimental data are available in the literature to identify potential links between thyroid function/diseases and PCOS. These findings include preclinical data on the effect of thyroid hormones on ovaries and impact of sex hormones on the thyroid, autoimmunity as a link between PCOS and thyroid dysfunctions, and influence of endocrine disruptors on PCOS. Due to the lack of epidemiological studies on the relationship between specific thyroid diseases, such as thyroid cancer and PCOS, indirect evidence was reported.

#### 3.3.1 Preclinical studies

Few preclinical data showed a strong interaction between hypothalamus-pituitary-gonadal and hypothalamus-pituitary-thyroid axes, suggesting an association between PCOS and thyroid disorders. Hypothyroidism in rats after thyroidectomy increased ovary size and the local concentration of luteinizing hormone (LH)/human chorionic gonadotropin receptors (45). Therefore, low levels of thyroid hormones appeared to sensitize the ovaries to gonadotropin action and favor the development of PCOS in rats (46). In an animal PCOS model (female Wistar rats treated with estradiol valerate) several biochemical and histological evidence of thyroid gland dysfunction was observed, such as increased TSH serum levels, decreased T3 and T4 serum levels, small-sized thyroid follicles devoid of the colloid and increased connective tissue between follicles (47). Interestingly, treatment of the rats with PCOS with chamomile extract and metformin improved serum levels of TSH, T3, and T4 and counteracted most pathological changes in the thyroid gland (48). These data suggested a link between insulin resistance, a milestone in the pathogenesis of PCOS and thyroid function.

In another preclinical model of PCOS comprising rats treated with letrozole, an increased thyroid weight was reported (49). Combination treatment of melatonin and T4 for rats with PCOS restored thyroid weight to values observed in control groups, suggesting an interplay between melatonin, the thyroid, and PCOS (49). Expression profiles for messenger RNA (mRNA) and long non-coding RNA (lncRNA) in ovarian tissues from letrozole-induced rats with PCOS and control groups were characterized via deep sequencing (49). Pathway analysis revealed that differentially expressed mRNAs were related to several pathways, including insulin resistance, steroid hormone biosynthesis, PPAR signaling pathway, cell adhesion molecules, adenosine mono-phosphate-activated protein kinase signaling pathway, and autoimmune thyroid disease (50).

Finally, prenatal glucocorticoid exposure of mice resulted in metabolic dysfunctions in adult mice, and the effects of 3-iodothyronamine (T1AM), a natural analog of T4 with distinct functional properties, was explored. T1AM administration induced a profound tissue-specific antilipogenic effect in liver and muscle tissue, while an opposing effect on the regulation of estrogenic pathways was observed in the ovary by upregulation of *STAR*, *CYP11A1*, and *CYP17A1* (51).

#### 3.3.2 Autoimmunity in PCOS and thyroid diseases

Emerging research showed a link between PCOS, low-grade inflammation, and autoimmune disorders, suggesting PCOS pathogenesis as an autoimmune disease (52–54). Indeed, several studies reported a higher incidence of autoantibodies, such as anti-histone, anti-double stranded DNA, anti-nuclear, anti-thyroid, anti-sperm, anti-ovarian and anti-islet cells autoantibodies in patients with PCOS (55–58). Although the exact reason for this association is not known, several mechanisms were suggested, including hormonal susceptibility in women with PCOS.

PCOS is characterized by a hormonal imbalance with an increase in LH/follicle stimulating hormone (FSH) ratio, hyperandrogenism, insulin resistance, a moderate increase in serum estradiol levels and a decrease in progesterone production (1). Serum estradiol levels were higher in anti-TPO Ab-positive women with PCOS than in anti-TPO Ab-negative women with PCOS, and anti-TPO Ab positively correlated with estradiol and estradiol/progesterone ratio (59). These data were recently confirmed in adolescent females with PCOS (60).

Estrogen receptors are present on several cells of the immune system. Although estrogens have a dichotomous effect on the immune system regarding their concentration and timing of exposure (61), several reports suggested an association between excess estrogen production and the incidence of autoimmune diseases, probably due to the stimulatory effects of estrogen on interleukin (IL)-4, IL-1, IL-6, and interferon-γ (62, 63) production. Furthermore, activation of estrogen receptor-α was found to promote autoimmune responses and autoantibody production through an increase of β cell activity and a decline of T suppressor cell activity (64). Conversely, androgens and progesterone exert immunosuppressive and anti-inflammatory effects. Indeed, progesterone receptor activation counteracts the effects of estrogen receptor-α, which supports the production of autoantibodies (61, 65, 66).

Therefore, in women with PCOS an increased estradiol/progesterone ratio due to anovulation and luteal insufficiency may be involved in stimulating the immune system and consequently in the AIT development. The immunosuppressive effect of androgens makes it more difficult to explain the potential role of hyperandrogenism in patients with PCOS in AIT development. Less severe cases of hyperandrogenemia and hyperandrogenism were observed in patients with PCOS and AIT versus patients with PCOS without AIT (67). A recent experimental study (68) demonstrated a change in the expression of T helper (Th)17 and T regulatory (Treg) cells with an imbalance of the Th17/Treg cell ratio in peripheral blood mononuclear cells from patients with PCOS and AIT versus patients with PCOS without AIT, which was significantly influenced by testosterone (68).

Another potential link between PCOS and AIT appears to be mediated through the tumor growth factor (TGF)-β pathway, a main regulator of immune tolerance, by stimulating Treg cells, a subpopulation of T cells that act to suppress the immune response, thereby maintaining homeostasis and self-tolerance. This effect may be modulated via the gene for fibrillin 3 (FBN3) that regulates the bioavailability of TGF-β (10, 69). The FBN3 genetic variant, *D19S884* allele 8, was strongly associated with risk of PCOS (70). Women with PCOS carrying allele 8 of *D19S884* in the FBN3 gene had significantly lower levels of TGF-β1, higher inhibin β levels, and higher Homeostatic Model Assessment of Insulin Resistance levels than women with PCOS without the same allele (71). Similarly, in patients with autoimmune hypothyroidism lower serum levels of TGF-β1 were reported versus healthy controls (72). Thus, TGF-β and Treg level reduction may favour autoimmune processes and it can be speculated that women with PCOS carrying allele 8 of *D19S884* in the *FBN3* gene who have lower levels of TGF-β1, are more susceptible to developing hypothyroidism than women with PCOS without allele 8 (10, 73). Genetic susceptibility was considered a major factor in AIT and PCOS development in > 70% of cases, as demonstrated by studies of families and twins (10).

Vitamin D is well known for its beneficial and protective effect on the immune system. Indeed, vitamin D deficiency and polymorphisms of the *vitamin D receptor* (*VDR*) gene was associated with an increased risk of developing several autoimmune diseases, including hypothyroidism (74, 75). Similarly, several meta-analyses reported on the association of *VDR* gene polymorphisms with PCOS risk (76–78). Vitamin D deficiency may have a role in the development of insulin resistance, metabolic syndrome and hyperandrogenism in women with PCOS (79, 80). Vitamin D deficiency was significantly associated with AIT in overweight and obese patients showing that obesity, frequently present in women with PCOS, is associated with lower vitamin D circulating levels (81). Interestingly, 25-hydroxy vitamin D levels were significantly lower in women with PCOS and AIT versus women with PCOS without AIT (82). Therefore, low levels of vitamin D provide another possible link between thyroid dysfunction and PCOS in women with PCOS.

#### 3.3.3 Endocrine disruptors

An interesting hypothesis linking PCOS with thyroid alterations considers the deleterious effect of the endocrine disruptors. Available data (83) seem to suggest that the same endocrine-disrupting chemicals may induce epigenetic alterations at the ovarian and thyroidal level inducing PCOS and thyroid dysfunction. Unfortunately, solid evidence supporting this hypothesis is lacking and needs to be validated in future studies.

### 3.4 Effect of thyroid function/dysfunction on PCOS features, and vice versa

AIT, in the general population, is associated with a euthyroid phase followed by SCH, which slowly progresses to overt hypothyroidism (84). It is not clear if the euthyroid phase in women with PCOS has the same length/duration compared with the general population or whether the progression to overt hypothyroidism is different in terms of its timing/incidence. Considering data on autoimmunity and on low-grade chronic inflammation in women PCOS (52, 85, 86), it may be possible to hypothesize shorter euthyroid and SCH phases in women with PCOS and AIT (87). A recent retrospective case-control study (35) showed that TSH levels, adjusted for age and BMI, were higher in women with PCOS resulting in a higher incidence of SCH versus control groups. Although serum TSH levels were related to PCOM but not to hyperandrogenism or ovarian dysfunction, the patient proportion with PCOS and a complete phenotype increased with higher serum TSH levels (35). Conversely, the proportion of patients with non-PCOM and non-hyperandrogenic phenotypes decreased with higher serum TSH levels (35).

In two meta-analyses of studies of AIT in women with PCOS (14, 15), the analyses were not conducted according to distinct PCOS phenotypes or the relationship of AIT with different PCOS features. However, both studies suggested no relationship between BMI and AIT in women with PCOS because no difference in BMI was observed in patients with PCOS with/without AIT (14, 15). Moreover, it is well known that increased BMI and obesity influence the severity of the PCOS (14, 15). Limited data have failed to establish the effect of AIT in women with PCOS on serum LH and FSH levels and on the LH/FSH ratio (14). But the presence of AIT/HT was associated with increased insulin resistance in women with PCOS (88).

A large cross-sectional study (87) of 600 patients with PCOS and 200 age-, BMI-, and AIT-matched women, demonstrated that serum TSH levels were directly associated with hyperandrogenism, and the hyperandrogenic PCOS phenotypes were more frequent in patients with higher TSH levels (> 2.5 mU/L). Also, a recent retrospective analysis (39) showed a higher incidence of thyroid diseases, including AIT, increased thyroid volume, and more frequent thyroid nodules >1 cm in women with the complete PCOS phenotype. These findings, however, were not confirmed by other authors (35). The relationship between serum concentrations of anti-TPO Ab and the ovarian reserve in different PCOS phenotypes was specifically investigated (89). Although no difference was observed among PCOS phenotypes, an inverse relationship between anti-TPO Ab and serum anti-Mullerian hormone (AMH) in women with PCOS was observed suggesting that the ovarian tissue may be more sensitive to AIT in women with PCOS (89). Differences in the ovarian reserve among different PCOS phenotypes may be not significant due to the small sample size.

Women with PCOS have increased AMH serum concentrations due to enhanced AMH production per follicle and, such as in PCOS patients with PCOM, to increased follicle number (90, 91). Thyroid dysfunction may affect ovarian AMH production and the ovarian reserve, influencing PCOS diagnosis and its characteristics. Levels of AMH were lower in women with PCOS and AIT versus patients with PCOS without AIT, and negatively correlated with anti-TPO Ab levels and AIT duration (92). Interestingly, the coexistence of anti-TG Ab did not influence findings (92). No effect of anti-TG Ab was detected on ovarian function (menstrual pattern) or biochemical/clinical hyperandrogenism (92). More recently, serum AMH levels were significantly higher in anti-TPO Ab-negative versus anti-TPO Ab-positive women, but it is important to note that patient age was significantly lower in anti-TPO Ab- negative versus anti-TPO- Ab-positive women, highlighting the influence of age on AMH levels (93). Conversely, other studies (58, 94, 95) reported high autoantibody levels against ovarian tissue in women with PCOS. In particular, the presence of thyroid antibodies in ovarian follicular fluid and their correlation with AMH serum levels were demonstrated (96).

Despite conflicting findings, the relationship between AMH and the thyroid was extensively studied in populations not selected for PCOS. A large cross-sectional study (97) on patients with normal, low, and high ovarian response, showed no relationship between serum thyroid hormone and AMH levels. These findings were subsequently supported (97–100), although, in 2021, a meta-analysis of 9 trials confirmed that AIT and hypothyroidism may affect the ovarian reserve (101). Serum TSH levels <3 mIU/ml were associated with better ovarian function (102–104), suggesting that SCH may influence ovulatory function and menstrual cyclicity, therefore SCH is a crucial diagnostic criterion for PCOS (1–6).

One confounding factor for the relationship between thyroid function and ovarian function/reserve is patient age since thyroid alterations increase and worsen with age. A more recent study (105) in infertile women, including patients with subclinical and clinical hypothyroidism, confirmed a relationship between TSH levels and the ovarian reserve in women aged >35 years only. Although these data are not in agreement with other studies (93, 101–104, 106), one unit increase in TSH level was associated with a 25% increased risk of an AMH level <1.1 ng/ml and an increase in serum TSH values greater than the cutoff of 1.465 mIU/L was associated with a decrease in ovarian function in these patients (105). More recently (106) a Chinese study demonstrated that the ovarian reserve may be affected only in women with TSH levels >2.5 mIU/L and an anti-TPO Ab >100 IU/ml.

**Figure 1:**
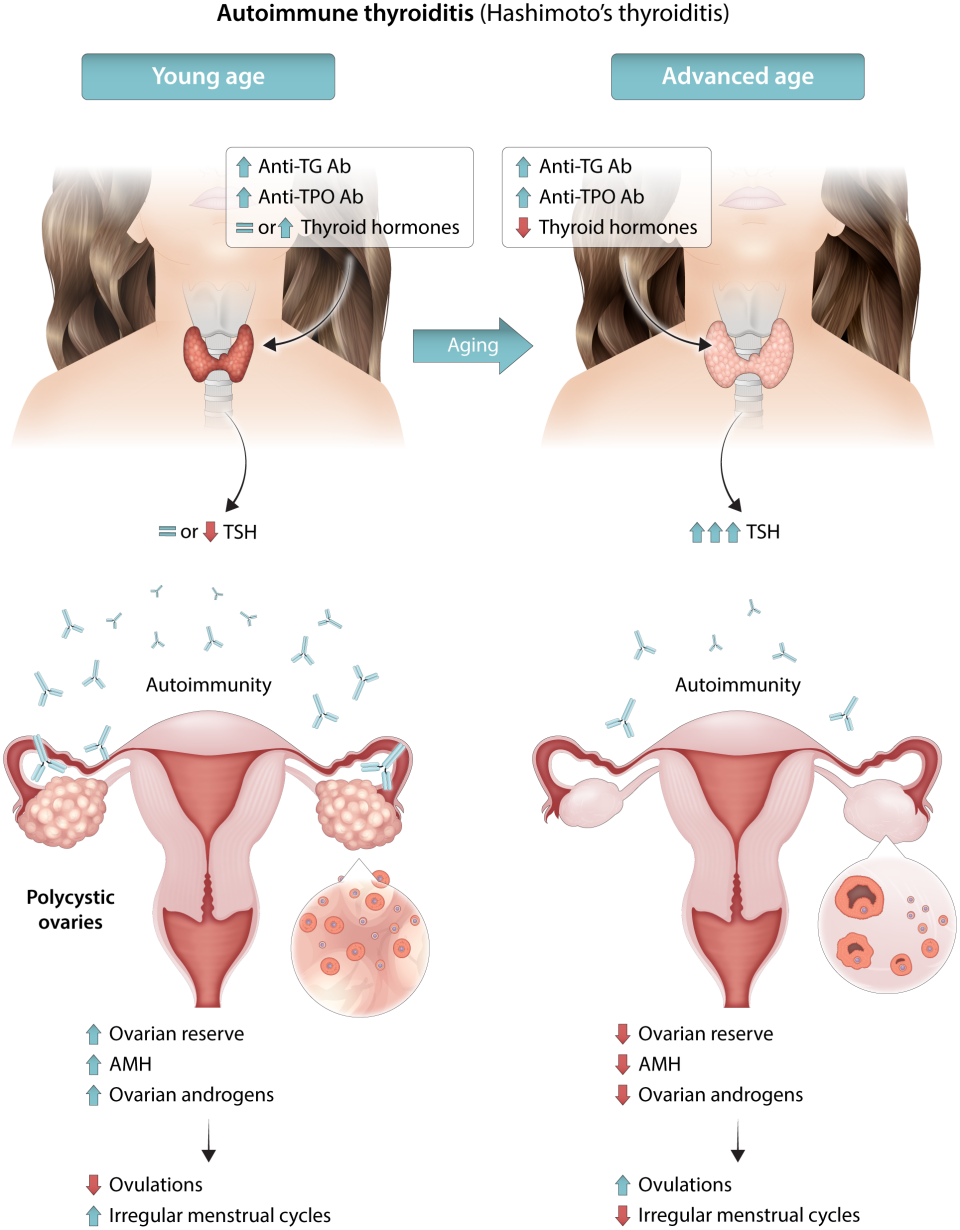
Schematic illustration for the possible mechanisms underlying the relationship between AIT, ovarian reserve, PCOS severity and phenotype. During the initial phases, the autoimmune thyroiditis may cause an autoimmune inflammatory process also involving the ovaries and may predispose young women to PCOS or more severe PCOS phenotypes. At later phases, with an increase in age, auto-antibodies may damage ovarian tissue, as well as the thyroid gland. The reduced ovarian reserve may induce milder PCOS phenotypes. AIT, autoimmune thyroid disease; AMH, anti-Mullerian hormone; anti-TG Ab antithyroglobulin antibodies; anti-TPO Ab, anti-thyroid peroxidase antibody; PCOS, polycystic ovary syndrome.

AIT coexistence in women with PCOS may also influence fertility (92). Thyroid hormones, irrespective of clinical dysfunctions, play an important role in the development and maintenance of reproductive function in women, directly affecting the ovary and endometrium via thyroid hormone receptors and indirectly affecting these tissues through the secretion of sex hormone-binding globulin, prolactin, and gonadotropin-releasing hormone (107). Hypo- and hyperthyroidism have adverse effects on female reproduction and are associated with a wide range of reproductive disorders, including abnormal sexual development, disturbances in menstruation and ovulation, and infertility (108). Furthermore, AIT presence in euthyroid women was associated with unexplained infertility, miscarriage, recurrent miscarriage, low fertilization rates, poor embryo quality in assisted reproductive technologies (ARTs), preterm delivery, and maternal post-partum thyroiditis (109–111). Anti-thyroid antibodies were detected in the ovarian follicular fluid of women with hypothyroidism at levels correlating with serum antibody levels, and their presence was associated with lower fertilization rates (96). PCOS was included among autoimmune-mediated disorders, such as endometriosis and premature ovarian failure (111–113). AIT presence was associated with unexplained infertility and implantation failure, irrespective of PCOS (114). Furthermore, the effects on AMH (92) may reduce the biological reproductive advantage of women with PCOS due to a higher ovarian reserve and/or larger reproductive window (115). In fact, exposure of the ovaries to autoantibodies may cause, similarly to thyroid gland, ovarian damage with reduced AMH levels (92). Several data demonstrated significantly higher levels of thyroid antibodies in infertile women with lower ovarian reserves (95, 116, 117), and this seems particularly true for anti-TPO Abs (98). From a clinical point of view, these data are particularly important for adolescent females and confirm the significant inverse relationship between serum AMH values and AIT (118). **Figure 1** summarizes the available data on the relationship between the ovarian reserve and AIT in women with PCOS.

Conversely, the potential influence of hyperandrogenism and other PCOS-related features, such as obesity and insulin resistance, on the thyroid gland is very complex. Experimental data suggest that sex hormones, specifically testosterone, may influence thyroid cancer initiation and progression (119). The presence of estrogen receptors in thyroid cancers (120) also suggests that estrogens may enhance thyroid cancer proliferation, whereas androgens and/or androgen receptors may play a protective role. However, the effect of androgens on thyroid cells seems to be gender specific. Testosterone levels in serum and thyroid cancer tissues were higher in women and lower in men with thyroid cancers versus related control groups, whereas binding activity and androgen receptor mRNA expression were enhanced in men and reduced in women with thyroid cancers (121). More recently, a 2.7-fold reduction in androgen receptor expression was detected in papillary thyroid cancer versus matched normal tissue (122). *In vitro* studies on undifferentiated thyroid cancer cells demonstrated that hormonal activation of androgen receptor with dihydrotestosterone addiction led to receptor translocation into the nucleus, proliferation reduction, and a shift towards G1 arrest (122). In contrast, a close and inverse relationship between thyroid nodules prevalence and serum sex hormone binding globulin (SHBG) levels was demonstrated in men, with a 1.91-fold lower risk in the lowest quartile of SHBG levels versus the highest quartile of SHGB levels (98).

Obesity, a crucial factor because it worsens the severity of PCOS (1), is related to a higher risk of thyroid cancer (123, 124), and is associated with several aggressive clinicopathologic features in both men and women (125). BMI was associated with extrathyroidal extension, multifocality, tumor size and lymph node metastasis in both overweight and obesity patients (125), although a linear association was demonstrated only in men (124). The potential role of obesity in thyroid cancer risk may also differ by gender or may be due to a more frequent abnormal metabolic pattern, including insulin resistance, altered adipocytokine profiles, and chronic low-grade inflammation in men versus women (126). Chronic low-grade inflammation, abnormal cytokine levels, insulin resistance, oxidative stress, and increased estrogen levels, frequently present in PCOS (1), are all factors that contribute to the occurrence and growth of thyroid cancer in obese patients (123, 127). Metabolic syndrome and its components are also associated with an increased risk of thyroid cancer, especially in obese patients and women (128, 129). However, for metabolic syndrome, obesity interaction with other metabolic components was different between men and women (128, 129). Similarly, metabolic dysfunction associated with alcoholic fatty liver disease was closely related to thyroid cancer in women (130).

Insulin resistance and hyperinsulinemia, crucial etiological factors for PCOS (1), are also well recognized risk factors for thyroid tumors (123, 131). In a multivariate analysis, insulin resistance was found to be an independent risk factor in euthyroid patients (132). Other PCOS-related comorbidities (1), such as dysglycemia, dyslipidemia, and hypertension, were risk factors for thyroid cancer (123). Abnormalities in the gut microbiota, frequently observed in PCOS (133), may represent a new link between PCOS and thyroid cancer (134, 135). Finally, patients with PCOS are frequently infertile (115) and infertility is also associated with a 29% higher adjusted risk of developing thyroid cancer (136).

In conclusion, several available data in the literature discuss how AIT potentially influences the ovarian reserve and its function, possibly modifying the PCOS phenotypes, and specifically increasing or reducing its severity according to patient age and the timing/evolution of the thyroid disease (**Figure 1**). Conversely, only indirect data partially support the role of PCOS-related features on thyroid function. Some PCOS-related features seem to be risk factors whereas others are protective factors. Current data may support the hypothesis that hyperandrogenism may play a protective and antiproliferative effect on thyroid cancer cells in women with PCOS. Moreover, PCOS-related hyperandrogenism may worsen the syndrome, increasing its severity and the prevalence of the several PCOS-related comorbidities, such as obesity and insulin resistance, that may counterbalance the overall risk of thyroid neoplasia.

### 3.5 Effect of thyroid alterations on cardiometabolic risk in women with PCOS

PCOS is closely associated with obesity, alterations in glucose and lipid metabolism, metabolic syndrome, and an increased risk for cardiometabolic disease (137). A large amount of data was available on the effect of high TSH levels and SCH in women with PCOS, whereas fewer studies analyzed the influence of other thyroid diseases/dysfunctions, such as AIT and GD, in women with PCOS. SCH was associated with a decrease in glucose disposal, but an increase in serum SHBG, total cholesterol (TC), low-density lipoprotein (LDL) cholesterol, and total triglyceride (TG) levels, weight, and insulin resistance in the general population, particularly in women (138–141). In fact, thyroid hormones regulate lipid and carbohydrate metabolism via direct actions on gene expression or nuclear receptors (142). In contrast, an interesting study (143) demonstrated that peripheral deiodinases and, consequently, serum FT3 concentration significantly decreased after glucose load in euthyroid women with PCOS versus age-matched, BMI-matched, and thyroid volume-matched women without PCOS. These changes were significantly related to insulin, TC, and TG levels in women with PCOS, suggesting that thyroid function alterations may affect metabolic patterns more greatly in women with PCOS versus those without PCOS (143).

These potential effects on metabolic and cardiovascular risk in the general population may translate to a significant increase in the categories who are at risk, such as those with obesity (144, 145), frequently associated with PCOS (1), and women with PCOS (137). The increase in cardiovascular risk due to SCH may be higher in obese patients with PCOS or specific subcategories of women with PCOS, such as women with the complete PCOS phenotype, hyperandrogenic phenotype, diabetes, or metabolic syndrome (146). For example, a large prospective cohort study (147) with a median follow-up of >17 years showed that there was a higher risk of cardiovascular mortality in diabetic patients with SCH versus the diabetic euthyroid control group (HR 1.52, 95% CI 1.08 - 2.12). Unfortunately, a subgroup analysis for patients with PCOS was not performed.

A systematic review with a meta-analysis (148) of 12 studies, including a total of 2341 women with PCOS (577 with SCH versus 2077 euthyroid control groups), concluded that SCH in women with PCOS does not influence the clinical profile (systolic and diastolic blood pressure, waist circumference, waist/hip ratio, and BMI), hormonal profile (prolactin, FSH, LH, LH/FSH ratio), and metabolic profile (serum SHBG and LDL cholesterol levels, and insulin resistance indexes) profile of women with PCOS (148). Only mild metabolic alterations were observed in women with PCOS and SCH comprising a lower serum high-density lipoprotein (HDL) cholesterol level (mean difference –3.92 mg/dL, 95% CI –6.56 to –1.29) and TG levels (mean difference 26.91 mg/dL, 95% CI -3.79 to 50.02) (148). Unfortunately, a large amount of heterogeneity was observed after data synthesis (148), probably due to the lack of predefined inclusion/exclusion criteria for PCOS and SCH in the study design.

In 2018, a meta-analysis (149), including 9 observational studies of 1838 women with PCOS (301 patients with SCH versus 1537 euthyroid control groups) demonstrated higher levels of TC, TG, and fasting glucose, lower levels of HDL, and no significant difference in serum testosterone levels in patients with SCH versus euthyroid control groups.

Another more recent systematic review and meta-analysis (150) analyzed 27 studies of 4821 women with PCOS, including 1300 patients with SCH and 3521 control groups without SCH. Data confirmed that patients with PCOS and SCH had a worse lipid profile and insulin resistance index than patients with PCOS without SCH, but serum testosterone levels were lower in patients with PCOS and SCH versus patients with PCOS without SCH (150).

In women with PCOS, serum TSH levels appeared to be strongly correlated with higher LDL cholesterol concentrations (151), TG, apolipoprotein B (apoB), and free testosterone, but was negatively associated with apolipoprotein A (apoA), independent of age, BMI, or thyroid autoimmunity (87). Furthermore, in women with PCOS, SCH was closely related to obesity (21, 152) and biochemical and clinical markers of insulin resistance (22) and worsened several features of insulin resistance versus euthyroid control groups (152). Data on the relationship between SCH hyperandrogenism is controversial (22, 150), and these findings are difficult to explain considering that SCH seems to generally worsen outcomes in patients with the PCOS phenotype and severity.

Although a large amount of data are available on the effect of high TSH levels/SCH on intermediate endpoints of cardiometabolic risk, limited data exists on the clinical risk to the patient. A large population-based study (24) showed that baseline TSH levels in women with PCOS was not predictive of later development of type 2 diabetes or cardiovascular disease. Data analysis on a large number of patients investigating the potential association between SCH and risk of developing diabetes also demonstrated no association, even after adjusting the data for age and sex (153). Unfortunately, no subgroup or sensitivity analysis for women with PCOS was performed. However, another large observational study (16) showed that the coexistence of AIT and PCOS greatly increases the coronary artery disease risk from ~50% for patients with AIT alone to approximately sixfold in patients with AIT and PCOS suggesting a role of AIT on the cardiometabolic risk in women with PCOS (irrespective of SCH), although the confidence interval was very large (16). In contrast, other authors reported that the coexistence of SCH and AIT did not clinically worsen metabolic alterations in patients with PCOS (22). Data analysis of a large number of patients including 38274 participants with SCH (154) demonstrated no difference in cardiovascular mortality for patients with AIT versus those without AIT (aHR 1.15, 95% CI 0.87−1.53). Therefore, the influence of AIT on the cardiovascular risk of patients with PCOS and SCH is not completely clear.

The relationship between SCH and PCOS in terms of cardiometabolic risk is very complex because it also involves clinical non-diagnostic characteristics influencing the severity of PCOS, and this is particularly true for obesity. Obesity is closely related to the elevation of proinflammatory markers and insulin resistance, and chronic low-grade inflammation, and is a crucial influencing factor of metabolic abnormalities in women with PCOS (1). In obese women, a relation between thyroxine deficiency and higher TSH levels due to decreased deiodinase-2 activity at the pituitary level via undetermined processes was suggested (155). Furthermore, leptin, a hormone produced primarily in adipose cells, that regulates energy balance by inhibiting or stimulating hunger, may stimulate the hypothalamus to secrete thyroid releasing hormone (TRH) in obese patients and influence the excess amounts of visceral adipose tissue through alterations in the hypothalamus-hypophysis-thyroid axis (156). However, TSH levels are also closely related to insulin resistance in women with PCOS, regardless of age or BMI, and TSH a cutoff of ≥2 mIU/L may be considered a specific predictor of insulin sensitivity in patients with PCOS (107). These findings were also confirmed in another study where women with PCOS and TSH levels < 2 mIU/L had significantly higher markers of insulin resistance compared with patients with TSH level ≥2 mIU/L (31).

In a large Taiwanese study (25), a higher incidence of cardiometabolic comorbidities, including hyperlipidemia, was detected for patients with GD and PCOS. In particular, the risk of hyperlipidemia, adjusted for confounders, was more than double in patients with GD and PCOS versus patients with GD without PCOS (aHR 2.18, 95% CI 1.14−4.17). For 6731 patients with AIT, including 3599 patients with GD and 1332 patients with HT, and 26924 patients in control groups, coexistence of PCOS and AIT was associated with a more than twofold higher risk of developing diabetes mellitus (aOR 2.48, 95% CI 1.14−5.38), hyperlipidemia (aOR 2.05, 95% CI 1.11−3.77), and coronary artery disease (aOR 2.63, 95% CI 1.06−6.51) in women with PCOS and AIT versus those with PCOS alone (25).

In conclusion, high TSH levels and SCH are associated with significant worsening of several intermediate endpoints of cardiometabolic risk in women with PCOS. However, the impact of SCH in women with PCOS appears to be low and it is not known whether SCH clinically influences long-term risk. The effects of SCH, alone or in association with AIT, in women with PCOS at high risk, such as obese prediabetic patients with severe phenotypes, should be investigated in the future. Data on other thyroid dysfunctions and PCOS are rarely reported in the literature and seem to suggest that cardiometabolic comorbidities may worsen when the two entities are present.

### 3.6 Effect of thyroid abnormalities on the reproductive outcome in women swith PCOS

Thyroid hormones play a crucial role in female reproductive system regulation (157). Overt thyroid dysfunction had a negative impact on both fertility and pregnancy outcomes (108, 109, 157–159). However, the effect of subclinical thyroid abnormalities on reproductive outcomes is much more debated. According to the most accredited theories, slight increases in TSH levels could exert a negative effect on both embryo implantation at the endometrial level and the fertilization process (160). Furthermore, anti-thyroid Ab, acting on granulosa cells, could further hinder the success of the reproductive process (161). Available data suggest a negative impact of SCH on obstetric and neonatal outcomes (162). Maternal SCH in pregnancy is a well-demonstrated risk factor for small babies, those at gestational age, and babies with a low birth weight, with a 24% higher risk in patients with SCH versus the euthyroid control group (163). But data regarding their effect on fertility-related outcomes are conflicting. Slight isolated elevations of TSH levels do not appear to have a negative impact on fertility and miscarriage risk (159). However, anti-thyroid Abs were associated with an increased rate of miscarriage and recurrent pregnancy loss (109, 164). Women with PCOS have reduced fertility overall (115) due to reduced oocyte (165) and endometrial (166) competence. These abnormalities may also influence the risk of early and late pregnancy complications in patients with PCOS. Several maternal and neonatal complications are increased in women with PCOS (167). Hypertensive disorders and gestational diabetes are more frequent in women with PCOS, even after adjusting the data for BMI (168, 169).

Thyroid disease risk during first birth and the one-year post-partum period in women with PCOS in Denmark was significantly increased versus patients without PCOS (OR 2.3, 95% CI 2.0 - 2.8) (24). The highest risk was reported for hypothyroidism (OR 3.0, 95% CI 2.3 - 3.9), although a significant influence on risk was also observed for the presence of a goiter (OR 2.0, 95% CI 1.1 - 3.4), thyrotoxicosis (OR 1.7, 95% CI 1.1 - 2.3), thyroiditis (OR 2.3, 95% CI 1.2 - 4.2), or postpartum thyroiditis (OR 1.3, 95% CI 0.7- 2.6) (24). Thyroid medication use was more than double in patients with PCOS versus patients without PCOS (OR 2.5, 95% CI 2.1 - 3.0), and the risk remained significant for both thyroid replacement treatments (OR 2.7, 95% CI 2.2 - 3.3) and anti-thyroid medications (OR 1.7, 95% CI 1.1 - 2.4) (24).

A recent cross-sectional study (170) analyzed biochemical thyroid parameters in 69 pregnant women with PCOS, 354 control groups, and the cord blood of their babies at birth. Serum thyroid level alterations were more prevalent among women with PCOS. FT3 levels were significantly lower in women with PCOS versus those without PCOS, whereas an increased anti-TPO Ab prevalence was observed in mothers and babies with PCOS versus those without PCOS (170). In patients with elevated anti-TPO Ab levels, hypothyroidism prevalence was higher in patients with PCOS versus patients without PCOS (170). However, no association between complication rate and thyroid parameters was found (170).

In conclusion, the combined effect of PCOS and thyroid dysfunctions, as well as their relationship, on reproductive performance are not still completely known. However, from available data, it can be speculated that mild thyroid dysfunctions may influence reproductive outcomes in women with PCOS, and vice versa, enhancing the deleterious effects of AIT/SCH or PCOS on reproduction. Thus, patients with PCOS and subclinical thyroid dysfunction may have a higher infertility rate due to ovulatory dysfunction/pregnancy loss, and pregnant women with PCOS and AIT may have a higher risk of developing SCH and overt hypothyroidism during the gestation period compared with women without PCOS. Furthermore, patients with PCOS and AIT/SCH may be considered a subpopulation at high risk of obstetric and neonatal complications. Women with both PCOS and SCH/AIT could represent a phenotype at a higher risk of reproductive dysfunction and, consequently, a target population for whom testing the effect of therapeutic interventions (e.g., levothyroxine) is needed. It should be noted that the efficacy of therapeutic interventions such as levothyroxine has never been convincingly demonstrated in populations not selected for SCH/AIT (171, 172). There appears to be an informal consensus recommending thyroid function evaluation in women with PCOS (21, 152) to enhance their reproductive and clinical pregnancy outcomes, however, this suggestion is not formally incorporated in any PCOS treatment guidelines.

### 3.7 Thyroid function and dysfunction in patients with PCOS undergoing fertility treatments

Fertility treatments may be an excellent model for studying the impact of thyroid dysfunction associated with PCOS on human reproductive health. The effect of this potentially detrimental combination on each stage of the reproductive process from oocyte fertilization to embryo implantation requires investigation. Since the exact biochemical stage of the pregnancy is precisely determined during fertility treatment, this enables investigation of the effect of AIT/SCH in women with PCOS at the earliest gestational stages (164). Obese women with PCOS should first be counselled on lifestyle modifications, such as a hypocaloric diet and physical exercise, before starting pharmacological treatments (6, 173, 174). If these interventions fail, and in anovulatory non-obese patients with PCOS, oral ovulation induction with letrozole or clomiphene citrate (CC) may be considered as first-line treatment (6). Gonadotropins can be considered as an alternative first-line treatment following counselling on the cost and potential risk of multiple pregnancy or can be prescribed as second-line agents in women who failed prior treatment with oral ovulation induction (6).

It is important to note that it has been hypothesized that AIT may negatively influence the outcomes of ovulation induction cycles with these pharmacological agents (95). Accordingly, patients with such disorders were excluded from all RCTs comparing the effectiveness of different treatments for ovulation induction in women with PCOS (173). Therefore, the impact of thyroid dysfunction on the success rate of ovulation induction cycles can only be extracted from the few retrospective data that are available (95). In a retrospective cohort study conducted on 196 infertile women with PCOS, anti-TPO Ab levels exceeding the upper reference limit were found in significantly more CC-resistant patients versus CC responders or metformin responders (95). According to the authors, anti-TPO Ab may therefore represent a new predictive marker of a response to oral ovulation induction (95). Similarly, SCH was also a predictor of CC resistance, although it is not known whether thyroid dysfunction exerts a direct effect on the ovary or an indirect effect, through increased insulin resistance or a metabolic syndrome (175). Unfortunately, to the best of our knowledge, those findings remain isolated, and this hypothesis still awaits validation.

In women affected by anovulatory infertility due to PCOS, *in vitro* fertilization (IVF) should be considered when ovulation induction cycles are unsuccessful or in the presence of concomitant factors of infertility, such as tubal damage or the male factor (6, 176). Meta-analyses (177, 178) showed that women with PCOS versus those without PCOS, had similar rates of clinical pregnancy and live births but, also achieved more unfavorable outcomes. Moreover, a reduced fertilization rate and an increased miscarriage risk was detected (177, 178).

The relationship between PCOS and AIT, as well as SCH, could be particularly critical in women seeking fertility treatments (10). A recent updated systematic review and meta-analysis showed that women with AIT undergoing ART cycles had a ~30% reduction in the rate of implantation, a ~50% increase in miscarriage risk, and a ~30% reduction in the chance of live births (179). Interestingly, these results did not appear to be influenced by age or serum TSH concentration. However, both associations were not observed in a subgroup analysis of patients who exclusively underwent intracytoplasmic sperm injection (179, 180), confirming previous findings from a meta-analysis (181). It is tempting to speculate a detrimental additive or, at least, synergic effect of PCOS and AIT on the miscarriage risk and, consequently, live birth rate but this remains undemonstrated. Furthermore, the studies included in the aforementioned meta-analyses investigating the impact of AIT on ART outcomes did not divide the data based on infertility etiology (179, 181). Furthermore, an adequately powered study on women with PCOS comparing reproductive outcomes between AIT-positive and AIT-negative patients has not been published. Therefore, AIT was not included in the most recent meta-regression analysis exploring the influence of possible covariates on the association between PCOS and miscarriage (182).

SCH was also hypothesized to have a negative effect on ART outcomes. To investigate this, a recent meta-analysis was performed to evaluate the association between preconception maternal TSH levels and IVF success rate (183). When the TSH cutoff value for SCH was set to 2.5 mIU/L, no significant differences were observed in any clinical reproductive endpoints between patients with SCH and the euthyroid control group. Notably, when a higher TSH level was used to diagnose SCH (i.e., 3.5–5 mIU/L), a significantly increased miscarriage risk was observed in affected women (183).

Based on this, it is possible to speculate that PCOS and AIT and/or SCH may represent a phenotype with a particularly poor reproductive prognosis during the IVF cycle. Initial indirect evidence can be extracted from studies investigating the independent impact of TSH level on IVF-related outcomes in women affected by PCOS (36, 184). A small prospective cohort study on 32 AIT-negative patients with PCOS and a serum TSH level of 0.4−4.5 mIU/L (184), showed that both serum and follicular fluid TSH levels were negatively correlated with the rate of oocyte maturation and fertilization, whereas high-quality embryo production rate was negatively correlated with serum TSH concentration only (184). Interestingly, the authors also observed an increased TSH receptor expression and cyclic adenosine monophosphate concentration in granulosa cells of patients with PCOS, suggesting a possible detrimental effect of TSH on the PCOS ovary mediated via TSH receptor/cyclic adenosine monophosphate (184) with a potential reduction of oocyte competence in women with PCOS (165). More recently, a multi-factor linear regression analysis was performed to identify variables that influence the maturation of oocytes in a cohort of 594 euthyroid women with PCOS selected for IVF (36). Serum TSH concentration was negatively correlated with oocyte maturation, and a TSH cutoff value of ≥2.98 uIU/ml was identified as a predictor of a poor oocyte maturation rate (36). Oocyte maturation rate was better in women with TSH levels <2.98 uIU/ml versus patients with higher TSH levels (36). Further prospective evidence is warranted to clarify the impact of TSH levels on the reproductive success rate of women with PCOS.

However, a change of perspective is needed. Available data strongly suggest that thyroid function should be considered a dynamic element in women undergoing ovarian stimulation (185). This could be particularly relevant for patients with PCOS with a greater risk of a high response to ovarian stimulation and, therefore, of hyperestrogenism, a supraphysiological condition that undermines thyroid function equilibrium (185). Future studies should, thus, consider assessing thyroid function immediately before embryo transfer. The eventual identification of a new TSH threshold associated with poorer reproductive outcomes would allow the design of interventional studies with a therapeutic goal of optimization of TSH levels after/during ovarian stimulation (185). Considering the quality of the available evidence, it is timely to investigate the impact of AIT on reproductive prognosis in women with PCOS. Furthermore, the impact of ovarian stimulation on AIT cannot be ruled out for women with PCOS (108). The ideal time to assess anti-TPO Ab and anti-TG Ab levels is at the end of the ovarian stimulation process. A very promising alternative study population includes women who undergo freeze-thawed embryo transfer cycles, even though this would not provide information on embryological parameters. The absence of the confounding effect of ovarian stimulation in this study population would enable the investigation of the impact of thyroid function and AIT, independently, on reproductive outcomes in women with PCOS.

### 3.8 Thyroid medications in women with PCOS

According to the American Thyroid Association guidelines, for hypothyroid women seeking pregnancy, the levothyroxine dose should be adjusted to achieve a TSH level between the lower reference limit and 2.5 mIU/L (186). Available evidence is insufficient to determine whether levothyroxine supplementation improves fertility in anti-TPO Ab-positive women with SCH who are attempting to have a natural conception (186). However, considering the effect of ovarian stimulation on the thyroid axis function and impact of thyroid dysfunction on fertility treatment outcomes, women undergoing ART should be considered a distinct population (180). The 2021 European Thyroid Association guidelines on thyroid disorders before/during ART recommend levothyroxine for women with AIT and TSH levels >2.5 but <4.0 mIU/L, with a low levothyroxine dose before ovarian stimulation, on a case-by-case basis (180). Specifically, the decision to treat patients with thyroid medications should consider the following concomitant factors: a diminished ovarian reserve/premature ovarian insufficiency; age >35 years; history of recurrent pregnancy loss; and anti-high thyroid Ab levels (180). PCOS was not included in this group of patients at increased risk due to insufficient data. However, women with PCOS, slightly elevated TSH values, and AIT may benefit from levothyroxine supplementation. Levothyroxine may at least partially restore mechanisms that have been altered by both PCOS and AIT/SCH. Firstly, levothyroxine may improve the ovulation process by exerting a positive effect not only on menstrual cyclicity, but also on oocyte and endometrial quality. Secondly, levothyroxine administration may induce an improvement in many risk factors for cardiovascular events and lead to a better reproductive outcome and long-term reduction in the cardiometabolic risk.

Although interesting and biologically plausible, these hypotheses are not supported by current evidence. A small case-control study (151) demonstrated no significant effect of levothyroxine treatment in women with PCOS and SCH after multivariate analysis; none of the serum metabolic markers assessed, including LDL and HDL cholesterol, triglycerides, glucose, and insulin changed after treatment. Similarly, an interesting study showed that the well-known positive association between number of live births and risk of autoimmune hypothyroidism is stronger in women with PCOS versus women without PCOS, despite the greater use of thyroid medication in women with PCOS (24). Thus, specific, robust data on the administration of levothyroxine in women with PCOS are urgently needed.

### 3.9 Effect of PCOS treatments on the thyroid

An interesting question is whether PCOS treatment can improve thyroid function. Many treatments for this are available for women with PCOS (**Table 4**). Obese patients should follow lifestyle modification programs, including improved diet and physical activity, to reduce body weight and improve insulin sensitivity (2–6). As previously mentioned, women with PCOS and ovulatory infertility should receive oral ovulation induction agents or gonadotropins with/without metformin (6). Metformin is also effective in improving menstrual cyclicity and intermediate cardiometabolic endpoints (6, 187). Oral contraceptives (OC) and antiandrogens also play a role in menstrual irregularities and establishing hyperandrogenism features (3–6). Many other treatments are also suggested.

**Table 4:** Key scientific evidence on the effect of treatments for PCOS on thyroid function and disease according to the Oxford Centre for Evidence-Based Medicine 2011 Levels of Evidence (http://www.cebm.net/index.aspx?o=5653).

PCOS treatment	Thyroid effect	Grade of evidence
Lifestyle modification programs (diet and physical activity)	Controversial data on thyroid hormones levels and development of thyroid cancer	Level 3
Metformin	Controversial data on potential reduction in serum TSH levels and thyroid nodule size	Level 2
Oral contraceptives and anti-androgens	Potential risk of hypothyroidism development Potential increased risk of thyroid cancer	Level 3
Vitamin D	Low vitamin D levels are potentially correlated with PCOS and AIT development	Level 2
Myoinositol	Improvement of SCH and AIT	Level 5

AIT, autoimmune thyroid disease; PCOS, polycystic ovary syndrome; SCH, subclinical hypothyroidism; TSH, thyroid stimulating hormone.

The effect of lifestyle factors and its modifications on the thyroid is controversial. A recent review (188) reported on the effect of smoking (that causes a decrease in TSH levels and an increase in T3), BMI (that has a direct relationship with TSH and free T3 levels), and iodine intake (that has a direct relationship with TSH levels and an indirect relationship with T3 levels) on TSH and thyroid hormones. Regular physical activity influences thyroid hormone levels, inflammation, and immune system markers (189) and may exert a beneficial effect on thyroid function. However, data on the effect of physical activity on the thyroid are also controversial. Some studies, including cross-sectional and longitudinal analyses, demonstrate no association between TSH/FT4 levels and physical activity (190), whereas others reported that more active adults tended to have lower TSH and T4 levels and a somewhat blunted TSH response to reduced T4 levels (189). Recently, no effect on thyroid function was observed following a three-week isocaloric ketogenic diet (191). An effect of physical exercise on thyroid cancer risk has been also suggested (190, 192, 193), however, in all studies the clinically meaningful effect was modest.

OC may potentially influence thyroid function by enhancing liver SHBG synthesis and modulating tissue estrogen action (194). Long-term OC use was associated with a ~4 times higher risk of hypothyroidism. Data from a study (24) with a large sample size also suggested that treatment with OC was an independent risk factor for the development of thyroid disease in women with PCOS, and this effect could be mediated by enhancing autoimmunity, inflammation, insulin resistance, and weight gain (24). A meta-analysis (195) demonstrated a small but significantly increased risk of thyroid cancer, especially for patients with the papillary histology, in OC users versus non-OC users. Progesterone administration also increased the thyroid cancer risk (196).

Data on the effect of infertility drugs on thyroid disorders, including cancer, are controversial (196), especially regarding the use of CC, particularly at high dosages and in nulliparous women, whereas the administration of gonadotropins was associated with an increased thyroid cancer risk (196).

Data on metformin administration on patients with PCOS are also limited. Metformin administration induced a significant TSH level decrease in women with PCOS and hypothyroidism (197, 198), irrespective of whether the patients received levothyroxine (198). In women unselected for PCOS, data from a meta-analysis (199) confirmed that metformin induces a significant reduction in serum TSH levels and thyroid nodule size suggesting a potential effect of metformin on TSH levels/the thyroid by improved insulin resistance. Patients treated with metformin have a smaller thyroid volume and a lower risk of incident goiter and thyroid nodules (200). The inhibitory effects of metformin on the thyroid appear to involve several pathways related to adenosine monophosphate-activated protein kinase, mammalian target of rapamycin, mitochondrial glycerophosphate dehydrogenase, and the nuclear factor κB (201). However, recent data on the effect of metformin on thyroid cancer risk are vague (202). Metformin induced a significant reduction in anti-TPO Ab and anti-TG Ab levels in patients with HT and SCH (203), reduced TSH levels, and increased FT4 and FT3 levels in euthyroid patients with uninodular thyroid disease and insulin resistance (204).

Post-hoc analyses of two randomized controlled trials (RCTs) (205) including 288 pregnant women with PCOS randomized to metformin or placebo from the first trimester to delivery showed that the overall prevalence of SCH (1.5%) and overt hypothyroidism (0%) was similar between two groups, and TSH level was not affected by metformin. Of note, metformin resulted in a significant decrease in observed FT4 levels throughout the pregnancy period, correlating with reduced weight gain and a nonsignificant lower prevalence of gestational diabetes (OR 0.85, 95% CI 0.71−1.02) (205).

Many studies have investigated the role of vitamin D in reproduction. Low vitamin D levels have been detected in women with PCOS (206) and patients with thyroid dysfunctions (207). Vitamin D levels were markedly lower in patients with AIT, HT, or hypothyroidism, but not in patients with GD (207). In women with PCOS, vitamin D levels were significantly lower in patients with AIT versus patients without AIT suggesting that low vitamin D levels may play a role in the pathogenesis of AIT in patients with PCOS (82). Consequently, vitamin D supplementation was proposed for several potential beneficial effects on metabolic and biochemical parameters (208, 209). In patients with AIT, vitamin D administration decreases serum anti-TPO Ab and anti-TG Ab levels (210). Unfortunately, studies assessing the effect of vitamin D supplementation in women with PCOS and thyroid dysfunctions, such as AIT, are not available. Moreover, interesting data demonstrate a synergistic effect of metformin and vitamin D in prediabetic women with HT (211).

Inositol is a cyclic polyol with 6 hydroxyl groups that may exist in 9 possible isoforms. Myoinositol was the first isoform identified and it is the most abundant in the eukaryotic cells (212). It’s clinical and metabolic efficacy for treating women with PCOS has been recently confirmed in meta-analytic studies (213). Recent papers (214, 215) reviewed the available *in vitro* and *in vivo* studies detailing the role of myoinositol on thyroid homeostasis and showing a potential favorable effect of myoinositol supplementation on SCH and AIT.

In summary, the effect of vitamin D deficiency on the pathogenesis of PCOS and thyroid dysfunctions and whether this impact is a consequence or coexisting factor needs further investigation in RCTs using standardized methods and specific definitions for vitamin D deficiency.

## 4 Discussion

### 4.1 Key findings

PCOS and thyroid disorders are two of the most common endocrine diseases affecting young women worldwide and contribute to severe metabolic and reproductive disorders. All available guidelines and consensus documents on PCOS diagnosis reviewed emphasized that PCOS is a diagnosis of exclusion for which diagnosis is possible only after excluding disorders that mimic the PCOS phenotype. Accordingly, published articles suggest excluding thyroid dysfunctions. However, these articles do not clarify which thyroid dysfunctions/diseases should be excluded or the tests and cutoff values to use. Current analysis demonstrates a higher prevalence of thyroid diseases, particularly AIT and SCH, in women with PCOS versus control groups, indicating a close association between thyroid disorders and PCOS. Conversely, data on clinical hypothyroidism, Graves’ disease/hyperthyroidism, goiter, thyroid nodules, and thyroid cancer in women with PCOS are limited. Similarly, direct evidence on the PCOS risk in populations with thyroid diseases are also scarce. Furthermore, many experimental data in animal models of PCOS suggest a strong relationship between thyroid function perturbations and PCOS and emphasize many potential and biologically plausible mechanisms for this, although the exact cause/underlying mechanisms for this association is not yet fully understood. Several mechanisms alone/combined, including sex hormone production, inflammation, and autoimmunity, may play a role in this for patients with a genetic susceptibility.

Direct and unequivocal evidence on the effect of thyroid function/disorders on PCOS features are lacking, and this impact may be confounded and biased by several factors. High TSH levels and SCH are associated with a substantial worsening of several intermediate endpoints of cardiometabolic risk in women with PCOS, although the impact of SCH in women with PCOS appears to be small and mediated by BMI and insulin resistance. Thyroid abnormalities may worsen reproductive outcomes, especially in patients undergoing fertility treatments, but direct evidence of this in women with PCOS are limited. To date, despite a clear rationale, there is no data demonstrating the efficacy of thyroid medications, particularly levothyroxine, on fertility and the cardiometabolic risk in women with PCOS. Lifestyle modification changes, including diet and physical activity, metformin treatment, and vitamin D supplementation appear to improve thyroid function in the general population and, thus, may be useful for women with PCOS, also.

### 4.2 Interpretation

The relationship between PCOS and the thyroid is complex because their separate characteristics can influence and be influenced by thyroid function and disease. This is true for diagnostic criteria, i.e., ovulatory dysfunction, hyperandrogenism, and PCOM, but also for nondiagnostic features closely related to the PCOS/thyroid function, such as obesity and insulin resistance. Furthermore, age appears to influence PCOS severity, as well as thyroid disease. The effects of AIT may vary depending on patient age; AIT may be associated with hyperthyroidism during the initial phases and SCH and clinical hypothyroidism at the later phases. Although a direct effect of AIT on the ovaries was not demonstrated in women with PCOS, it is plausible that in women with PCOS and AIT, particularly in presence of anti-TPO Ab, autoantibodies pass through the blood-follicle barrier during follicular evolution. During the initial phases of the thyroid disease, the disease may cause an inflammatory process involving the ovaries that stimulates AMH production and predisposes young women to PCOS or severe PCOS phenotypes. At later phases, with an increase in age, thyroid antibodies may damage ovarian tissue, as well as the thyroid gland, and ovarian reserve. Therefore, the presence of AIT may induce severe or milder PCOS phenotypes according to phase of disease or age (**Figure 1**).

Furthermore, available literature indicates that BMI and insulin resistance are factors that strongly modulate the effect of thyroid function/dysfunction on PCOS, and vice versa. Thus, obese insulin-resistant patients with PCOS may have an elevated risk of coincidental or future thyroid disease.

### 4.3 Strengths

In this narrative review, significant effort was made to interpret the main data available on the relationship between thyroid function and PCOS, and vice versa, to discuss the topic in a comprehensive fashion. An extensive literature search was performed using many keywords associated with PCOS features such as specific thyroid diseases. We analyzed experimental data to define the scientific plausibility of a potential connection between these endocrine diseases and the clinical evidence from epidemiological and intervention studies. Studies on patients with PCOS with the highest evidence were preferentially included, but when specific data for selected patients with PCOS were absent, data from studies that included general/unselected patient populations with specific characteristics (e.g., obesity) were also discussed.

### 4.4 Limitations

A wide variation in findings across studies was observed. This was particularly true for the incidence of thyroid diseases in patients with PCOS. This was probably due to the differences in the criteria used for diagnosis of PCOS and thyroid disease, particularly for AIT and SCH. Diagnostic criteria for PCOS were not specified in many studies. Other potential confounders influencing the findings were differences in the size of study populations, ethnic origins, geographical locations, anthropometric parameters, and iodine nutrition levels. Another limitation was that many studies had a short-term follow-up period. This was particularly true for SCH and PCOS, which frequently co-occur and share many endocrine and metabolic characteristics. However, findings from studies in patients with PCOS and SCH appeared to be conflicting in terms of reproductive and cardiometabolic effects, most likely because large data on selected populations and long-term follow-up were lacking.

Exclusion of thyroid diseases for a PCOS diagnosis is of interest but seems not to have been considered in many studies. In a registry-based study on 18476 women with PCOS diagnosis according to Rotterdam criteria and 54757 age-matched controls, a high incidence of thyroid diseases, including overt hypothyroidism and hyperthyroidism/thyrotoxicosis, was detected in patients with PCOS (24). This observation seems to suggest that in many retrospective/cohort studies the PCOS diagnosis, even if performed according to specific and well-recognized criteria, such as the Rotterdam criteria, is wrong or, at least, not entirely correct.

### 4.5 Implications for clinical practice

Coexistence of PCOS and thyroid diseases may identify patients with a more aggressive phenotype in terms of their reproductive and metabolic risk. Regular screening for thyroid function and thyroid-specific autoantibodies in women with PCOS, particularly before/during pregnancy, should be highly recommended. Furthermore, it can be hypothesized that levothyroxine is beneficial in obese/insulin-resistant patients with PCOS and SCH, together with medications for cardiometabolic alterations e.g., metformin for thyroid function (especially for patients with PCOS and glucose intolerance/diabetes mellitus) or vitamin D supplementation for vitamin D deficiency.

### 4.6 Implications for future research

To date, it is not recommended that women with PCOS are monitored for thyroid diseases, even though a large amount of data appear to recognize that these women have a high risk of thyroid dysfunction. Similarly, there is no clinical/scientific recommendation for the management of subclinical/clinical thyroid diseases in patients with PCOS under specific clinical conditions. Should the recommendations be more stringent in specific cases, e.g., for infertile patients or pregnant women with PCOS? Should PCOS diagnosis be confirmed also in presence of subclinical thyroid disease? If so, for which PCOS phenotypes? For example, considering the strong relationship between thyroid function and menstrual cyclicity, PCOS may be confirmed only for patients with the normo-ovulatory PCOS phenotype. More data are also needed on reference ranges for thyroid hormones in women with PCOS and on how to define a patient who has thyroid disease but is euthyroid under treatment with the PCOS phenotype. Is it important to consider these two diseases as independent comorbidities or should the PCOS diagnosis be excluded? Evidence-based answers for these questions are still needed.

## 5 Conclusions

Thyroid diseases and PCOS seem to be associated by their prevalence in experimental studies, but more comprehensive investigations are needed to fully understand their relationship in terms of their etiology, pathogenesis, and clinical outcomes. Questions remain unanswered on whether PCOS leads to AIT/SCH (or vice versa), whether treating patients for PCOS reduces their risk of thyroid dysfunction, and whether levothyroxine treatment in women with PCOS and SCH improves their reproductive/cardiometabolic outcome. However, regular screening for thyroid function and thyroid-specific autoantibodies in women with PCOS, particularly before/during pregnancy, should be highly recommended.

## Author contributions

SP conceptualized and designed the study, acquired, and interpreted the main data, and drafted the article. CC acquired the main data and additional references and drafted the article. AB, DC and GV checked the literature searches, improved the interpretation of data, and critically revised the article. All authors provided their approval of the final version for publication and agree to be accountable for all aspects of the work, especially regarding its accuracy and integrity.
